# Therapeutic Potential of Thymoquinone in Glioblastoma Treatment: Targeting Major Gliomagenesis Signaling Pathways

**DOI:** 10.1155/2018/4010629

**Published:** 2018-01-31

**Authors:** Fabliha Ahmed Chowdhury, Md Kamal Hossain, A. G. M. Mostofa, Maruf Mohammad Akbor, Muhammad Shahdaat Bin Sayeed

**Affiliations:** ^1^Department of Clinical Pharmacy and Pharmacology, University of Dhaka, Dhaka 1000, Bangladesh; ^2^Department of Pharmaceutical Chemistry, University of Dhaka, Dhaka 1000, Bangladesh

## Abstract

Glioblastoma multiforme (GBM) is one of the most devastating brain tumors with median survival of one year and presents unique challenges to therapy because of its aggressive behavior. Current treatment strategy involves surgery, radiotherapy, immunotherapy, and adjuvant chemotherapy even though optimal management requires a multidisciplinary approach and knowledge of potential complications from both the disease and its treatment. Thymoquinone (TQ), the main bioactive component of* Nigella sativa *L., has exhibited anticancer effects in numerous preclinical studies. Due to its multitargeting nature, TQ interferes in a wide range of tumorigenic processes and counteract carcinogenesis, malignant growth, invasion, migration, and angiogenesis. TQ can specifically sensitize tumor cells towards conventional cancer treatments and minimize therapy-associated toxic effects in normal cells. Its potential to enter brain via nasal pathway due to volatile nature of TQ adds another advantage in overcoming blood-brain barrier. In this review, we summarized the potential role of TQ in different signaling pathways in GBM that have undergone treatment with standard therapeutic modalities or with TQ. Altogether, we suggest further comprehensive evaluation of TQ in preclinical and clinical level to delineate its implied utility as novel therapeutics to combat the challenges for the treatment of GBM.

## 1. Introduction

Glioblastoma multiforme (GBM) is a primary neuroepithelial tumor of the brain, characterized by an aggressive clinical phenotype derived from inter- and intrapatient genomic and histopathological diversity [1]. In the latest reclassification of the World Health Organization (WHO), the GBMs are listed in the group of diffuse astrocytic and oligodendroglial tumors reflecting their highly malignant behavior [2]. It constitutes more than 40% of all malignant brain tumors and approximately 54.4% of all malignant gliomas with mean age at diagnosis being 64 years and 1.5 times more common in men than women [3]. Even after the treatments by multimodal therapy that involved surgery, radiotherapy, and combined chemotherapy, GBM is nearly incurable with approximate survival rate of around 8 to 15 months after diagnosis [4]. Genomic analysis for prognostic markers of GBM has been conducted with large-scale genomic characterization. These investigations found mutations or amplifications of different signaling pathways [5–8]. The most commonly disrupted signaling cascades in GBM are pathways related to receptor tyrosine kinase (RTK), including epidermal growth factor receptor (EGFR), platelet derived growth factor receptor alpha (PDGFRA), basic fibroblast growth factor receptor 1 (FGFR-1), and insulin-like growth factor receptor (IGFR-1) [9], and nuclear factor-*κ*B (NF-*κ*B) [10]. GBM has also been associated with aberration in signaling through the mitogen-activated protein kinase (RAS/MAPK), phosphatase inosine 3 kinase/protein kinase B (also known as AKT)/mammalian target of rapamycin (PI3K/AKT/mTOR), cell cycle-regulating retinoblastoma (RB) tumor suppressor related pathways, tumor protein p53 (TP53) [11], promoter methylation of O-6-methylguanine-DNA methyltransferase (MGMT), isocitrate dehydrogenase (IDH) mutation, and altered expression of cyclin dependent kinase (CDK) genes [12].

The logical leap from such investigation is that targeting disrupted pathways may be an effective means of treating GBM as is the case for other type of cancers [13]. The changes in RTK, PI3K, TP53, cell cycle, neoangiogenesis, cellular metabolism, NF-*κ*B [10], signal transducer, and activator of transcription 3 (STAT3) [14, 15] signaling pathways have already paved the way for considering them as feasible targets in GBM [10, 16, 17]. Among the RTKs, the instance of increased gene copy number of EFGR is prevalent in GBM, which is frequently responsible for increased proliferation, transformation, adhesion, migration, and escape from apoptosis [18]. Though extensive preclinical studies with GBM have shown promising results in EGFR targeting, several clinical trials designed for therapeutic targeting of EGFR in GBM patients have failed so far [19]. The lipid kinases PI3K family are situated downstream of RTKs and with the interaction of numerous intermediary signal transduction kinases (e.g., Akt, PTEN, and mTOR), control protein translation, ribosome biosynthesis, and cell growth [20, 21]. Targeting PI3K, PTEN, and mTOR pathways have shown moderate success in combination with conventional therapy at clinical level [17, 22, 23]. Alterations in cell cycle regulatory signaling pathways, for example, CDK signaling (especially mutation of CDK4, CDK6, and CDKN2A followed by E2F1 transcription factor dysregulation), and inactivation of TP53 (either dependent or independent of MDM2 mutation), have also been extensively targeted in GBM [8, 24]. Moreover, some studies with cell cycle inhibitors in GBM therapy have shown promise in radiosensitization as well as in the promotion of senescence and apoptosis of tumor cells [25, 26]. Neoangiogenesis, a characteristic histopathologic feature of GBM, is in part secondary to the hypoxic tumor microenvironment that induces hypoxia-inducible factor-1*α* (HIF-1*α*) followed by subsequent VEGF accumulation, RTK activation, fibroblast growth factor (FGF), PDGF, hepatocyte growth factor (HGF, also known as scatter factor), integrins, angiopoietins, and STAT3 upregulation [14, 27]. Depriving cell of oxygen and nutrients to halt further growth is the initial justification for targeting angiogenesis. The Food and Drug Administration (FDA) of the United States of America has already approved a monoclonal antibody targeting VEGF (Bevacizumab) in GBM therapy [4] but other interventions targeting angiogenesis have not shown improvement in overall survival in GBM [28, 29]. Clinical trials targeting multiple players in the angiogenesis pathways in GBM are underway but the outcomes are yet to be published [17]. The mutation of IDH (a component of the tricarboxylic acid cycle) occurs early in the gliomagenesis, leading towards neoenzymatic activity that converts *α*-ketoglutarate to 2-hydroxyglutarate and disrupts cellular metabolism in GBM [30, 31] providing rationale for considering IDH as a therapeutic target and it has already prompted several clinical trials whose outcome is yet to be available [30, 32, 33]. Glioma stem cells (GSCs) represent another viable target in GBM treatment due to their important role in mediating therapeutic resistance [34]. A number of other novel targets, such as poly-ADP ribose polymerase (PARP) (DNA repair protein), BRAF (a protein kinase that mediates MAP kinase signaling), bone-marrow X-linked kinase (BMX), Bruton's tyrosine kinase, and gamma-secretase, are now under investigation for GBM treatment at preclinical level[17, 35–37]. However, chemotherapeutic drugs still remains the mainstay in glioblastoma treatment. At present, the chemotherapeutic drugs for GBM approved by FDA act as alkylating agents (Temozolomide (TMZ) and Nitrosourea) [4, 38] which are not sufficient to combat GBM. Based on this situation, there have always been a need to find new therapeutics for GBM.

Thymoquinone (2-methyl-5-isopropyl-1, 4-benzoquinone; TQ) is the principle active ingredient of the volatile oil of black cumin or black seed (*Nigella sativa* L. (NS)) (family Ranunculaceae) [39]. People in different societies used NS as condiment and different traditional medicinal system such as Ayurvedic and Unani systems consider NS for the treatment of various maladies [40–43]. The pharmacological investigations of TQ [44] are almost as old as its isolation from NS in 1963 by El-Dakhakhny [45]. Since then, numerous preclinical studies have been performed including those to determine the anticancer effects of TQ. The molecular mechanism of through what TQ shows selective cytotoxicity for human cancer cells is widely reported [46]. Studies have shown that TQ causes selective cancer cell death and possess tumor growth inhibitory activities in addition to its role in interference with other tumorigenic processes such as angiogenesis, invasion, and metastasis [47, 48]. TQ is involved in tumorigenesis or development of drug resistance [49] as well as in the sensitization of cancer cells to chemotherapeutic agents and radiation therapy through the resistance mechanisms [31, 50]. The result of a registered investigation for studying the role of NS in a precancerous disease, actinic keratosis (AK), is yet to be reported (ClinicalTrials.gov Identifier: NCT01735097; website: https://clinicaltrials.gov/ct2/show/record/NCT01735097 accessed on 26 June, 2017). Hence, the usefulness of TQ in cancers including GBM is now more than a speculation and it can target different hallmarks [51] of GBM.

There are several reviews on GBM [52], its pathology [53, 54], possible therapeutic targets [17, 55, 56], and current challenges in its therapies [54]. The treatment with TQ alone has shown antitumor efficacy in several* in vitro* and* in vivo* studies [57, 58] and also as in adjuvant therapy either to prevent carcinogenesis [59] or to potentiate the efficiency of conventional therapeutic modalities [60]. However, there is no systemic compilation of the potential role of TQ as a therapeutic agent or as an adjuvant agent for the treatment or the prevention of GBM or an agent for slowing the progress of GBM. In this review, we have compiled the potential role of TQ in GBM therapeutics focusing on the major gliomagenesis signaling pathways.

## 2. Potential Role of TQ in Modulating Proliferative and Migratory Signaling Pathways of Glioblastoma

Two of the most important signaling cascades frequently deregulated in GBM are the PI3K/Akt/mTOR and Ras/Raf/MEK/ERK pathways that promote cell growth and proliferation [61, 62]. In addition, the dysregulation of RTK, non-RTK (c-src activity), growth factors (e.g., PDGF, FGF, TGF, and IGF-1), GTPase activating protein (G protein), serine/threonine-specific protein kinase (STK), and NF-*κ*B activity differentially contributes to GBM proliferation [63–66]. Studies have shown that aberrant constitutive activation of NF-*κ*B, in response to PDGF overexpression/PI3K signaling/PTEN inactivation, can promote GBM proliferation through inappropriate activation of regulatory genes that control cell proliferation and cell survival [67]. Nonreceptor tyrosine kinase, Focal Adhesion Kinase (FAC), is associated with increased rates of both migration and invasion in GBM [68]. The FAC signaling regulates cell adhesion and motility by relaying extracellular matrix (ECM) signals to ERK signaling and secreting matrix metalloproteinase- (MMP-) 2 and MMP-9 [69–71].

**Figure 1:**
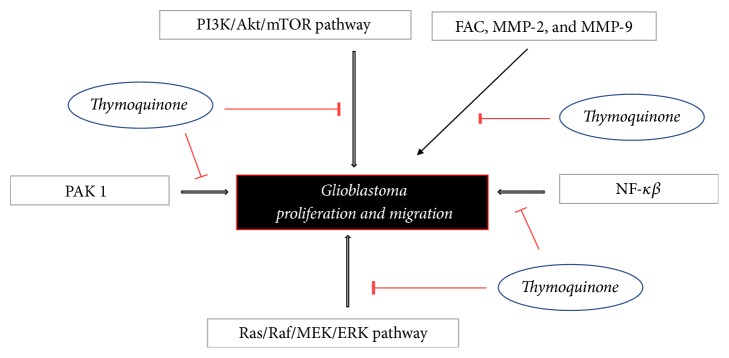
*Signaling pathways of proliferation in glioblastoma targeted by Thymoquinone *(NF-*κβ*: nuclear factor-*κβ*; FAC: Focal Adhesion Molecule; MMP-2: Matrix Metalloproteinase-2; MMP-9: Matrix Metalloproteinase-9; PAK1: p21 protein (Cdc42/Rac) activated kinase 1; PI3K/AKT/mTOR: phosphatase inosine 3 kinase/protein kinase B (also known as AKT)/mammalian target of rapamycin. Ras/Raf/MEK/ERK: a chain of proteins in the cell that communicates a signal from a receptor on the surface of the cell to the DNA in the nucleus of the cell; Ras: a type of small GTP-binding protein; Raf: Raf kinase family of serine/threonine-specific protein kinases; MEK: a protein kinase; ERK: a member of the mitogen-activated protein kinase superfamily). Aside from FAC, MMP-2, and MMP-9, other signaling pathways primarily affect glioblastoma proliferation.

No study has been conducted yet regarding the role of TQ in modulating the proliferative signaling pathways in GBM but studies in other type of cancer have demonstrated that TQ upregulates PTEN signaling [72, 73], interferes with PI3K/Akt signaling and promotes G(1) arrest, downregulates PI3K/Akt and NF-*κ*B and their regulated gene products, such as p-AKT, p65, XIAP, Bcl-2, COX-2, and VEGF, and attenuates mTOR activity [73–78], providing the strong rationale that TQ might play a crucial role in inhibiting PI3K/Akt/mTOR signaling pathways, NF-*κ*B, resulting in inhibiting proliferative signaling pathways of GBM. Studies in colorectal cancer have demonstrated that TQ inhibits the Ras/Raf/MEK/ERK signaling and disrupts its prosurvival function, especially affecting the kinase domain of the p21 protein (Cdc42/Rac) activated kinase 1 (PAK1), consequently disturbing its interaction with pPAK(Thr423) [79]. Phosphorylated Pak1 level in the cytoplasm has also been reported to correlate with shorter survival time in patients with GBM [80]. Multiple studies have reported that TQ downregulates FAC and reduces the secretion of MMP-2 and MMP-9 and thereby reduces GBM cells migration, adhesion, and invasion [81, 82]. Therefore, there is a strong possibility of TQ to provide therapeutic benefits for the treatment of GBM (Figure 1). However, we propose further investigation in this regard.

## 3. Cytotoxic and Antiapoptotic Potential of TQ against Glioblastoma

**Figure 2:**
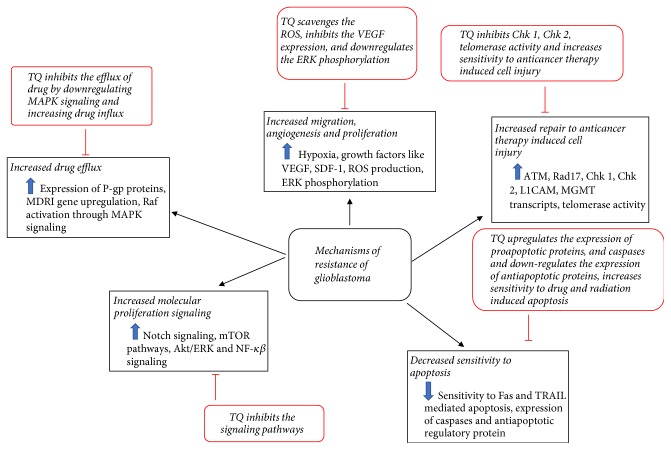
Antiapoptotic and chemosensitization potential of Thymoquinone.

TQ may exhibit glioma cell-specific cytotoxic effects [83] by influencing cell cycle, DNA structure and synthesis, structural proteins like tubulin, apoptotic mechanism, and ROS generation (Figure 2). It has been reported that TQ can interfere in normal cell cycle progression and thereby inhibit GBM growth [84]. Several studies have shown that TQ has the capacity to cause cell cycle arrest at different phases [46, 85]. TQ treatment can alter the expression of multiple cell cycle regulatory proteins, such as cyclin D1, cyclin E, and the CDK inhibitor p27 [74], and induce apoptosis (accumulation of sub-G1 population) through caspase activation and PARP cleavage [86].

TQ influences both p53-dependent and p53 independent pathways for apoptosis [72, 87]. TQ augments the proapoptotic and reduces the antiapoptotic regulatory proteins. TQ induced apoptosis involves changes in mitochondrial membrane potential, activation of caspases and PARP cleavage [88], increase in the Bax/Bcl-2 ratio via downregulating Bcl-2 and upregulating Bax level [88], raise in level of cytochrome c and caspase-3, along with suppressed expression of Bcl-xL and survivin [74], degradation of alpha and beta tubulin, and increase in p73 expression leading to apoptosis in cancer cells [87].

TQ is hypothesized to act as an antoxidant at lower concentrations and a prooxidant at higher concentrations depending on its environment [89]. In tumor cells specifically, TQ generates ROS production that leads to reduced expression of prosurvival genes, loss of mitochondrial potential, and structural changes in proapoptotic changes causing caspase-dependent apoptosis in the cells [90]. A recent study on human colon cancer cells demonstrated elevated level of ROS generation and simultaneous DNA damage when treated with a combination of TQ and artemisinin [91]. TQ mainly attenuates its proapoptotic and oxidative potential through suppressing the NF-*κ*B pathway [92]. This simultaneously inhibits the activation of IKBA kinase, IKBA phosphorylation, IKBA degradation, p65 phosphorylation, and p65 nuclear translocation. The expressions of NF-*κ*B-regulated antiapoptotic (IAP1, IAP2, XIAP Bcl-2, Bcl-xL, and survivin), proliferative (cyclin D1, cyclooxygenase-2, and c-Myc), and angiogenic (matrix metalloproteinase-9 and VEGF) gene products are also downregulated by TQ [92, 93].

TQ affects the DNA structure by targeting the copper in the chromatin, which is associated closely with the base guanine [89]. In normal cells, DNA damage will initiate repair by p53 mediated p21 triggered growth inhibitory effects. However, in GBM cells, TQ induced DNA damage directly causes cell death [94]. DNA-dependent protein kinase (DNA-PKcs) is necessary for repairing breaks in DNA double strand in order to maintain genomic integrity [95]. However, despite the prominent cell damage in DNA-PKcs deficient GBM cells, they are found to be less sensitive to TQ induced cytotoxicity as compared to DNA-PKcs proficient GBM cells. The significant cell death seen in DNA-PKcs proficient GBM cells justifies the theory that these cells are attacked by TQ and that DNA-PKcs activation is essential for cellular death in GBM [96].

Telomere attrition, due to inhibition of telomerase by TQ through the formation of G-quadruplex DNA stabilizer, subsequently leads to rapid DNA damage which can eventually induce apoptosis in cancer cells specifically [97]. In a recent GBM cell line study, TQ has shown to reduce telomerase activity and cause significant DNA damage [94] in addition to its inhibitory role in DNA synthesis in cancer cells affecting cellular proliferation and viability [58].

## 4. Targeting Chemosensitization and Drug Resistance Mechanisms of Glioblastoma by TQ

GBM possesses very complex resistance mechanisms associated with cell cycle and DNA repair, apoptosis, drug efflux, growth factors, and cellular maintenance pathways [98, 99]. Evidently, TQ can significantly affect the drug resistance of GBM through inhibition of its resistance strategies and induce chemosensitization, by acting as an adjuvant to a therapy via affecting variety of signaling pathways (Figure 2). GBM stem cells are seen to demonstrate an increased sense of repair to injury after radiotherapy, due to enhanced activation of ATM, Rad17, Chk1, and Chk2 [100]. Another cell surface protein molecule known as L1CAM, which is expressed nearly twice in resistant GBM cells, amplifies the DNA repair capacity through adhesion [101]. There is a 32-fold increase in the level of the repair enzyme MGMT transcripts in GBM cells which acts as a means of resistance against the anticancer alkylating agents [102]. TQ is seen to reduce the level of Chk1 (cell cycle checkpoint kinase) in p53^−/−^ HCT116 colorectal carcinoma cells. As a result this increases the caspase 3 activity leading to DNA damage and apoptosis, decreasing the extent of DNA repair [103].

GBM cells have decreased sensitivity to both Fas-mediated and TRAIL-mediated apoptosis [104, 105]. The reduced presence of caspase-8 [106] and increased expression of Bcl-2, an antiapoptotic regulatory protein [107], are the contributory causes behind the resistance. In a study with a model of colorectal tumorigenesis, it was observed that TQ increases the chemosensitivity of 5-fluorouracil (5-FU) by suppressing the NF-*κ*B pathway and upregulating antitumorigenic proteins [108]. Telomerase and DNA-PKcs deficiency play huge role in cellular resistance to apoptosis of GBM cells [94, 109]. However, GBM cells also carry specific mutations and miRNAs that inactivate the apoptosis [107] for what further investigations are required regarding the relevance of TQ treatment.

The higher apoptotic index in GBM is supported by their higher proliferation, presence of hypoxic region, angiogenesis, and migration [107]. The cancer cells facing hypoxia tend to remain inactive, do not proliferate, and create resistance to the cytotoxic anticancer drugs which cannot reach those [110, 111]. Extensive research has elucidated the fact that cancer cells express higher level of hypoxia-inducible factors [112]. Hypoxia induces the production of ROS that favors the tumor survival, progression, and adaptation [113]. TQ acts as an antioxidant in this case and scavenges the ROS including the superoxide anion, hydroxyl radical, and singlet molecular oxygen [114]. It has the capacity to readily travel across the blood-brain barriers (BBB) and reach the subcellular compartments [115], thus reaching the inner hypoxic regions of the tumor in the brain.

The mTOR is a protein kinase that ensures supply of nutrients to tumor cells and inhibits apoptosis and autophagy. This kinase is upregulated in tumors causing enhanced growth and proliferation, through Akt signaling [20]. The Notch signaling is another pathway found enhanced in tumor cells, which is necessary for the activation of transcription factors required for regulation of nervous system. Its activity is mediated by an enzyme gamma-secretase that stimulates its active signaling [116]. However, gamma-secretase activity can be inhibited by reducing Akt activity [117]. Thus both mTOR and Notch pathways involve Akt/ERK signaling that is downregulated by TQ [77]. TQ was found to chemosensitize gemcitabine against cancer cells in inducing apoptosis by inhibition of Akt/mTOR/S6 signaling pathways and reduced expression of antiapoptotic proteins [118], providing a strong rationale for the potentiating role of TQ with gemcitabine in GBM therapy [119].

Active efflux of anticancer drugs out of the cancer cell by ATP-binding cassette (ABC) transporters is one of the major criteria of resistant glioblastoma cells [120]. Overexpression of P-glycoprotein (P-gp), an ABC transporter, occurs because of the upregulation of the MDR1 gene, which is induced by mutation, activation of Raf, anticancer drugs, and DNA damaging agents [121]. Although TQ cannot prevent drug efflux directly, but it can indirectly help through inhibition of Raf activation, by downregulating MAPK [122], which will consequently downregulate the MDR1 gene expression, thus preventing the overexpression of P-gp. TMZ is currently the most widely used chemotherapy for GBM [123]. TQ is seen to give synergistic effect with TMZ in inducing apoptosis and cell growth inhibition in GBM cells [124]. As TQ is a small lipophilic molecule and, as mentioned earlier, can easily cross the BBB, it can help the chemotherapeutic agent in reaching the tumor while being used as an adjuvant and also by preventing the drug efflux indirectly, creating chemosensitization.

## 5. Potential Role of TQ to Mediate Neuroinflammation and Immunotherapy in Glioblastoma

GBM plays role in generating immunosuppressive microenvironment by producing different immunosuppressive cytokines including IL-6, IL-10, and TGF-*β* as well as tumor aggravating IL-1 and basic fibroblast growth factor (bEGF) resulting in neuroinflammation [125–127]. These cytokines promote antitumor immune response by inhibiting effector T cell response and activating regulatory T cell (Tregs) expression [128, 129]. In addition to cytokines, macrophage and myeloid derived suppressor cells also infiltrate into the GBM microenvironment and cause inhibition of antitumor immune response [130, 131]. Also, immunosuppressive checkpoints including CTLA-4, PD-1, LAG-3, and TIM-3 is known to have potential role in escaping immune environment of GBM [132].

Successful immunotherapeutic approach depends on its targeting of GBM cells specific antigen and its ability to kill tumor cells [129]. There are a number of antigens found that are glioma associated such as EPhA2, HER-2, gp 100, and TRP-2 [133–135]. However, EGFR found in around 25 percent of GBM patients is the most targeted one to the researchers. Peptide based vaccines for GBM usually target antigens such as EGFRvIII, survivin, and heat shock protein and currently couple of vaccines are in various stages of clinical trial including CDX-100 and M57-KLH [136]. Studies have provided evidence that TQ has potential to downregulate tumor associated antigen [137] and therefore hold promise to possess therapeutic benefit in inhibiting GBM antigen expression but further investigations are required in this regard.

Dendritic cells (DCs) vaccination is an important avenue of immunotherapy that utilizes DCs to make a bridge between innate and adaptive immune responses [138, 139]. Since DCs cannot process antigen effectively in immunosuppressive microenvironment, they are being cultured outside of patient body with exposing to antigen. Reinvigorated DCs are then inserted back to patient body which then activate T cells like CD4, CD8, and natural killer cells [129, 140]. Even though the DC based vaccine appears to be comparatively safe, its efficacy and clinical output are still limited. However, positive immune response among GBM patients were found when they are treated with DCs pulsed with different antigens with different degree of success [141, 142]. Studies have demonstrated that TQ compromises inflammation induced DC maturation, an important step towards antigen presentation to T cell and for effective antitumor immunity [143], and blunts inflammation induced cytokine release and migration of DCs [144], providing avenue for the further investigation regarding the role of TQ in DC cell based GBM therapy.

Immune stimulatory adjuvant are thought to initiate innate immune response through activation of toll-like receptors (TLRs) and pattern recognition receptors (PRRs), necessary components for maintaining the balance between both cellular and humoral immune response [145]. Commonly used adjuvants include CpG oligonucleotides, poly-ICLC, and tetanus toxoid. In two separate phase II clinical trials among GBM patients, it has been found out that treatment with poly-ICLC was well tolerated and improves the efficacy of radiotherapy [146, 147]. Studies have shown that TLR mediated by Neu1 sialidase activation [148] which is mediated by TQ provides avenue for further investigation regarding the role of TQ in the TLR mediated beneficial effect in GBM immune adjuvant therapy [149].

Immunomodulatory cytokines play important role in GBM and other cancer types. IL-6 is recognized for stimulating tumor growth in GBM patients, whereas IL-10 is known for inhibiting IFN-*γ* and TNF-*α* production [150, 151]. IL-10 is also responsible for decreased expression of MHC class II and inducing anergy in T cells [152, 153]. TGF-*β* is known to mediate immunosuppression by regulating T cell proliferation, IL-2 production, and NK cell activity and promoting regulatory T cells (Tregs) activity [154, 155]. A phase I clinical study among malignant glioma patients revealed that inhibiting Tregs by basiliximab, a monoclonal antibody to IL-2 receptor, generates improved immune response [56, 156]. TQ is known to inhibit the ability of TNF-*α* to induce IL-6 production in a different disease group [157]. This implies that further investigation is necessary to find out the impact of TQ on immunomodulatory cytokines in GBM patients.

**Figure 3:**
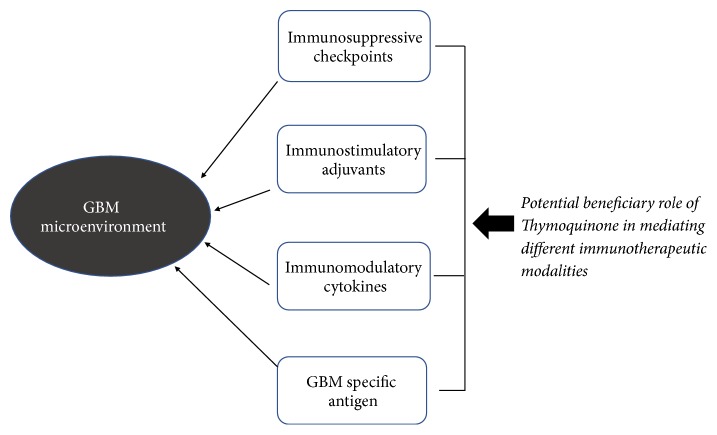
Potential beneficiary role of Thymoquinone in mediating different immunotherapeutic modalities.

TQ possesses significant antineuroinflammatory effect [158] and improves the anticancer activity of other therapeutic agent through either inhibition of autophagy or apoptotic cell death of GBM cell line [124, 159]. We hypothesize that TQ might help overcome those immunosuppressive mechanisms [160–162] in GBM immunotherapy and therefore further investigations are required for the potential role of TQ in different immunotherapeutic modules including the potential synergistic role of TQ on the therapeutic efficacy of immune checkpoints (CTLA-4 and PD-1) blockers for the treatment of GBM (Figure 3).

## 6. Potential Role of TQ to Inhibit GBM Stem-Like Cells from Acquiring a Mature Postmitotic Phenotype and Decrease Survival

It has been widely suggested that the subpopulations of tumor-initiating or stem-like cells are one of the primary factors causing GBM recurrence and resistance to treatment [100, 163]. It has been observed that GBM stem-like cells have higher levels of nuclear p65 and NF-*κ*B-dependent gene expression than regular glioma cells [164]. Studies have suggested that NF-*κ*B signaling has been linked to the proliferation, migration, and differentiation of neural stem cells [165] which is considered as one of the potential cell of origin of brain tumors. One of the subunits of NF-*κ*B, RelB, is highly expressed in mesenchymal GBM and studies have shown RelB regulates expression of Olig2 [166], a critical factor in normal and tumorigenic stem-like cell proliferation. Studies have shown that TQ interferes with the expression of RelB [167] and thus shows potential to interfere with tumorigenic stem-like cell proliferation. Studies have suggested that activation of NF*κ*B may keep differentiating glioblastoma-initiating cells (GICs) from acquiring a mature postmitotic phenotype, thus allowing cell proliferation and support the rationale for therapeutic strategies aimed to promote premature senescence of differentiating GICs by blocking key factors within the NF*κ*B pathway [168]. It is well established that TQ blocks NF-*κ*B from multiple molecular pathways but further investigation is suggested for the role of TQ in promoting senescence of GICs. Studies have shown that STAT3 is upregulated in GBM-derived brain tumor stem cells (BTSCs) [169] and inhibition of STAT3 either by pharmacological agent or by gene knockdown resulted in reduced BTSC survival regardless of endogenous MGMT promoter methylation or EGFR, PTEN, and TP53 mutational status [170]. TQ has shown to suppress STAT3 in myeloma, gastric, and colon cancer [86, 171, 172] and, therefore, we hypothesize that TQ would be inhibiting BTSCs but further investigations are warranted. We also propose more investigations regarding the role of TQ in preventing treatment resistance mediated from GBM stem-like cells in conventional GBM therapy.

## 7. Potential Beneficiary Role of TQ in Surgery and Ionization Radiation Therapy in Glioblastoma

Surgery and radiation therapy are two major modules for GBM treatment. The underlying molecular mechanisms that are overactivated or inactivated nearby the surgical area of GBM is still poorly understood [173] which partly contribute to the GBM reappearance, aggressive proliferation, and induction of metastatic potential in the microscopic tumors that are not eliminated through tumor resection [174, 175]. Many mitogenic and proangiogenic factors, such as TGF-*β*, FGF, VEGF, EGF-like growth factors, and endostatin, were found in the wound fluids that stimulate cancer cell proliferation and neoangiogenesis during postsurgery wound healing period [176–178]. Studies have shown that TQ attenuates tumorigenic signaling, including those mediated by TGF-*β*, VEGF, EGF, and several other promitogenic, angiogenic, and metastatic factors, with the inhibition of cancer cell growth, migration, and invasion [48, 179–182]. Studies have shown that TQ counteracts the trauma-induced chemotaxis of circulating malignant cells and their epithelial to mesenchymal transition (EMT) [48, 181, 183] and interferes in the activation of nuclear factor erythroid-related factor-2 (Nrf-2), NF-*κ*B, and STAT-3 that are responsible for the transcriptional activation of genes encoding proteins involved in cell proliferation, angiogenesis, and metastasis [50, 184]. Thus, TQ demonstrates very strong rationale for possible beneficial agent as a preoperative and/or postoperative neoadjuvant in GBM treatment.

Radiation therapy for GBM has been used in conjunction with surgery for over 35 years [185] and almost 50% of all cancer patients receive this therapy in one form or another during their course of illness [186]. It causes cell to undergo apoptosis due to double-stranded breaks via inducing DNA damage. Even though it is an effective therapeutics, it is restricted by some inherent limitations, such as the detrimental effect to surrounding normal tissues and the stimulation of cancer cells adaptive responses to counteract the damage process. Cancer cells that survived after initial cycles acquire resistance through multiple cellular mechanisms such as activation of NF-*κ*B, PI3K, Akt, and mTOR [187] but the resistance to radiotherapy in GBM is primarily attributed to EGFRvIII. This mutation confers an EGF ligand-independent dimerization of the EGF receptor resulting in constitutive activation of the EGF/EGFR signaling pathway [188, 189] and thus cellular resistance to radiation therapy by upregulating the DNA double‐stranded break repair machinery [190]. Therefore, EGFRvIII inhibitors are readily rationalized to possess increased overall GBM sensitivity to radiation therapy. Studies have found that EGFRvIII mutant GBM cell proliferation is more sensitive to TQ than wild-type GBM cells. It was also found that TQ inhibits autophagic flux and induces caspase-independent apoptotic cell death of the EGFRvIII mutant GBM cells to the similar extent of the wild-type GBM cells [115]. TQ might enhance radiation therapeutic benefit by enhancing the cytotoxic efficacy of radiation through modulation of cell cycle and apoptosis [31], preventing the radiation-induced metastatic progression through restoration of TGF-*β* [179] and activation of several signal transduction pathways including PI3K-Akt-mTOR [49, 187, 191, 192] or by rescuing T-lymphocytes from gamma irradiation-induced apoptosis [193]. Even though free radical scavenging ability and antioxidant properties of TQ are primarily considered for the mechanistic explanations of TQ mediated beneficial effect [192] but it is obvious that other mechanisms are involved and thus we propose further extensive investigations.

## 8. Summary and Future Perspective

GBM is one of the least understood diseases. Highly heterogeneous cell populations and complex pathogenesis add further complexities for effective therapeutic agent developments. The presence of BBB adds another layer of complexity in combating this disease. Though considerable advancements have been accomplished in GBM molecular pathogenesis and thereby in treatment strategies, the overall survival rates remain poor. Targeting a particular molecule or signaling pathway, involved in one of the singular aspects of the multistep complex tumorigenesis processes, has recently been deemed as extravagant attempt to curtail malignant progression. Due to the inherent heterogeneous nature, GBM can always evade a particular therapeutic modality and continue to survive on alternative pathways followed by recurrence of tumor at a far more aggressive form. Therefore, the paradigm in cancer treatment strategy is now shifting from targeted therapy to combination or multitargeted approaches or targeting cancer with modalities that affect multiple signaling pathways.

As a multitargeting therapeutic substance, TQ has been investigated in numerous disease models along with different types of cancer* in vivo and in vitro* models including GBM. Studies have focused on various signaling pathways providing evidence for its potential use in the GBM therapeutics. The prominent GBM signaling pathways includes the role of TQ in interfering in the phosphorylation and subsequent activation of several upstream tyrosine kinases (e.g., MAPK, Akt, mTOR, and PIP3) that are involved in tumor cell proliferation signaling pathways [49, 180]. Transcriptional factors (e.g., Nrf2, NF-*κ*B, and STAT-3) that are considered as key players in various oncogenesis process are other crucial molecular targets of TQ [49, 57, 84]. It has been suggested by multiple studies that, by regulating the activation of these transcription factors, TQ might counteract different tumorigenic processes including inflammation, cell proliferation, cell survival, angiogenesis, cell invasions, and metastasis. Furthermore, TQ shows chemopreventive properties by downregulating carcinogen metabolizing enzymes (e.g., CYP 1A2 and CYP 3A4), upregulating cytoprotective enzymes (e.g., glutathione S-transferase, superoxide dismutase, and oxidoreductase), attenuated production of proinflammatory mediators (e.g., cytokines, chemokines, and prostaglandins) [49]. Among different signaling pathways several are significant in the context of GBM therapy with TQ; the JAK/STAT and NF-*κ*B are getting increasing attention in the context of GBM. The JAK/STAT signaling in GBM consists of four JAKs (JAKs1–3 and TYK2) and seven STATs (STATs1–4, 5a, 5b, and 6) [194] but STAT3 is generally considered as the most eminent among cancers [195]. In GBM, protein kinase C*ε* has been shown to drive serine phosphorylation of STAT3 in a RAK/MEK/ERK-dependent fashion, and this modification of STAT3 enhances the invasive capacity and apoptosis resistance of GBM [196, 197]. STAT3 upregulation, hyperactivation, and nuclear accumulation is a well-known feature of GBM [198]. Studies have shown that TQ inhibits proliferation in gastric cancer via STAT3 pathway in vivo and in vitro [171] alone and also in combination with other drugs in breast cancer [199]. We propose further investigation for the role of TQ in GBM in JAK/STAT3 pathways. Further investigations are also required whether TQ affect specific parts of NF-*κ*B such as I*κ*K complex that is involved in the regulating NF-*κ*B activation or regulate NF-*κ*B signaling in a more selective manner by specifically interacting with NF-*κ*B dimers or whether TQ blocks NF-*κ*B by directly targeting the subunits (p65 and p50) themselves. This is of particular interest because of the fact that one available drug, TMZ, has opposite effects in the subunits [200]. Previous study in GBM cells has shown that Ikk inhibitors decrease proliferation and increase apoptosis directly [201] or via inhibiting nuclear p65 translocation [202]. Study regarding the effects of TQ on proteasomes is also suggested since inhibitors of proteasomes has shown to have beneficiary effects on GBM [203] but whether such effects are mediated by TQ has not been investigated.

TQ induces selective and time-dependent proteasome inhibition, both in isolated enzymes and in GBM cells, suggesting that this inhibition leads to intracellular increases in the levels of apoptotic proteins such as p53 and Bax, and may be linked to the onset of apoptotic events [204]. Therefore, we propose further investigations on TQ as its potential application as an adjuvant in the treatment of cancer and other diseases. In the clinical settings, no such study has been conducted with TQ for the treatment of GBM but one general conclusion is that improved understanding of the molecular mechanism by which GBM is regulated is a strategy that can make a significant impact in the successful management of GBM.

Interestingly, even the lower efficacy [205] and poor bioavailability [206, 207] of TQ are the primary bottleneck of TQ, its volatile nature [208] provides opportunity to be exploited for use in novel drug delivery strategy via intranasal pathway to brain due to unique connection provided by the olfactory and/or trigeminal nerve system present between the olfactory epithelium and the central nervous system. Such delivery system provides opportunity to bypass both the BBB and hepatic first-pass metabolism [209].

It is evident that TQ is multitargeting in its nature but majority of the signaling pathways in the GBM pathogenic context is yet to be explored. Due to its lower efficacy and systemic bioavailability, we propose further investigation on its role as adjuvant therapy with other chemotherapeutic courses. Further investigation could also be conducted for its more efficacious analogues and formulating those into different delivery systems to cross BBB in GBM treatment along with determination of their pharmacokinetic behavior, efficacy, and toxicity. To better understand these differential cellular effects of TQ, more in vitro, in vivo, and in silico studies could be conducted at both proteomic and genomic level. Findings from such studies will enable us to devise clinically effective combination therapeutics where TQ or its derivatives can potentiate the antitumorigenic potential of various conventional and established GBM therapeutic courses.
